# Pathways in Agro-Industrial Waste Upcycling: A Review of Sustainable Textile Innovations and Economic Perspectives

**DOI:** 10.3390/plants14233574

**Published:** 2025-11-22

**Authors:** Marina Proença Dantas, Carlos Rafael Silva de Oliveira, Natália Ueda Yamaguchi, Afonso Henrique da Silva Júnior, Rosane Marina Peralta, Adelar Bracht, Rúbia Carvalho Gomes Corrêa

**Affiliations:** 1 Graduate Program in Clean Technologies, Cesumar Institute of Science, Technology and Innovation-ICETI, Cesumar University, Maringá 87050-900, Brazil; marina.dantas@unicesumar.edu.br; 2 Graduate Program in Nanoscience, Processes and Advanced Materials (PPGNPMat), Federal University of Santa Catarina-UFSC, Blumenau 89036-004, Brazil; carlos.oliveira@ufsc.br; 3 Graduate Program in Energy and Sustainability (PPGES), Federal University of Santa Catarina-UFSC, Araranguá 88905-120, Brazil; natalia.ueda@ufsc.br (N.U.Y.); afonso.ufsc@gmail.com (A.H.d.S.J.); 4 Chemical Engineering Department, State University of Maringá-UEM, Maringá 87020-900, Brazil; 5 Graduate Program in Biochemistry, State University of Maringá-UEM, Maringá 87020-900, Brazil; rosanemperalta@gmail.com (R.M.P.); abracht@uem.br (A.B.)

**Keywords:** agri-food waste, circular economy, plant residue, sustainable manufacturing, textile processes, textile products

## Abstract

The growing concern over the environmental impacts caused by plant agriwaste has intensified the search for sustainable alternatives in manufacturing processes. This review explores the valorization of agro-industrial residues, such as those derived from banana, coconut, and pineapple, for example. It highlights their potential to be converted into value-added products, particularly within the textile sectors. Emphasis is given to the environmental and economic benefits of reusing biomass rich in fibers and bioactive compounds while discussing key technological, regulatory, and logistical barriers that still limit large-scale applications. In parallel, it presents recent advances in processing technologies, such as biocomposites and biochar, and the integration of circular economy principles to promote resource efficiency and waste reduction. The analysis also underscores the importance of public policies and financial incentives to drive innovation and ensure the viability of sustainable practices in industrial contexts. The article proposes an ideal circular production flow model that contrasts current linear practices with a regenerative, bio-based alternative. By mapping current challenges and future perspectives, this review expects to contribute to the debate on environmental responsibility, green technologies, and the economic potential of plant residue reuse in manufacturing chains.

## 1. Introduction

The textile industry is one of the most resource-intensive and environmentally burdensome sectors globally, characterized by substantial consumption of water, energy, and non-renewable raw materials, as well as the discharge of toxic effluents and solid waste [1]. The emergence and dominance of fast fashion, defined by accelerated production cycles, low-cost garments and short product lifespans, has intensified these challenges, fostering a culture of disposability that exacerbates textile waste generation and environmental degradation [2,3]. Synthetic fibers, particularly polyester, account for over 50% of global textile fiber production and are derived from fossil feedstock [4]. Their non-biodegradable nature contributes substantially to the persistent microplastic contamination problem in terrestrial and marine environments [5].

Furthermore, textile manufacturing, dyeing and finishing stages are among the most polluting industrial processes, producing high loads of chemically complex wastewater. If untreated, these effluents pose serious ecological and public health risks [1]. The prevailing linear economic model, sorted as unsustainable, demands a systemic shift toward circularity. This suggests rethinking material lifecycles through strategies such as design for longevity, resource recovery and waste valorization [6].

In parallel, the agro-industrial sector produces vast quantities of plant-based residues, including lignocellulosic waste, fruit and vegetable peels, seed husks, and fibrous stalks. Globally, agricultural and food industries generate over 1.3 billion tons of biomass annually, much of which is underutilized or incinerated, contributing to greenhouse gases (GHGs) emissions and environmental degradation [7]. However, this biomass is inherently rich in cellulose, hemicellulose, lignin, secondary metabolites, and other molecules that offer abundant and renewable possibilities for developing sustainable inputs for the textile industry.

Upcycling agro-industrial waste into value-added textile components represents a viable pathway to reduce the environmental footprint of both sectors. Technological advances now enable the transformation of such residues into regenerated fibers, natural dyes, biocomposites, and biodegradable auxiliaries, supporting both ecological sustainability and material innovation [2,8]. Notably, Clark (2022) emphasizes the role of green chemistry in designing benign processing methods that facilitate the integration of bio-based inputs without compromising performance [4]. Moreover, novel studies, such as the characterization of tucum (*Bactris setosa* Mart.) fibers, highlight the mechanical and morphological properties of underexplored native species, reinforcing the potential of regional agro-waste valorization for niche textile applications [9].

Beyond environmental imperatives, such upcycling approaches offer socio-economic advantages. They can stimulate rural economies by creating new value chains and employment opportunities in biomass-rich regions while reducing reliance on virgin textile resources [6,7]. As global awareness grows regarding the social and ecological costs of conventional fashion, consumer and policy-driven momentum aligns with the principles of a circular bioeconomy.

Although a growing number of studies address the valorization of agro-industrial waste, a comprehensive review focusing on its economic and sustainability aspects within the textile industry is still lacking. Previous reviews have typically focused on either specific residues, such as banana waste [10], or singular applications, such as natural dyeing [11], textile wastewater remediation [12,13], and the development of nonwovens [14] or cellulose fibers [15]. This leaves a gap in the literature for a study that connects these diverse innovations—spanning fibers, dyes, biocomposites, and auxiliary agents—and integrates a critical analysis of their technical and economic viability within a circular economy framework. Accordingly, the aims of this review are: (1) to map the progress over the last decade (2015–2025) in upcycling plant-based agro-industrial waste for the textile industry; (2) to evaluate the corresponding economic and sustainability challenges and opportunities; and (3) to propose an ideal circular flow model that incorporates these innovations.

## 2. Methodology

This narrative review was conducted through a structured literature search to map the advancements in the utilization of plant-based agro-industrial waste in the textile industry. The primary search was performed in May 2025 across the Web of Science (WoS) database, targeting the period from January 2015 to May 2025 to encompass the last decade of scientific and technological progress. The main search string combined the following descriptors: [(“agro-industrial waste” OR “agricultural residues” OR “plant residues” OR “biomass waste”) AND (“recycling” OR “reuse” OR “valorization” OR “recovery” OR “upcycling”) AND (“textile industry” OR “textile fibers” OR “textile materials” OR “textile inputs” OR “fibers”)]. The initial search yielded 213 publications. These results underwent an initial screening by title and abstract to verify their alignment with the scope. The initial inclusion criteria focused on original research and review articles that directly addressed the upcycling of plant-based waste for textile applications. The pre-selected articles were then read in full to confirm their relevance and to extract information. Finally, the reference lists of selected articles were manually reviewed to identify additional pertinent studies. Exclusion criteria included studies on non-plant or animal-based materials, works lacking experimental or analytical evidence and non-peer-reviewed publications. To reduce selection bias, two authors independently screened and evaluated the articles, resolving discrepancies by consensus. At the end of this multi-step process, the identification of the most significant documents occurred, which formed the basis for the critical analysis and synthesis presented in this manuscript.

## 3. Agro-Industrial Waste

In 2022, agricultural activities worldwide totalized 3.8 trillion dollars in added value and produced nearly 10 billion tons of commodities [16]. Sugar cane, maize, wheat and rice alone accounted for half of this production. In the same year, the agricultural sector consumed 3.7 million tons of pesticides [17] and 185 million tons of inorganic fertilizers [18], occupied 4.78 billion hectares of land [19] and maintained 892 million employers worldwide [20]. Furthermore, for most countries, agriculture consumes almost 90% of all water withdrawals [21].

Crop cultivation is essential to the global food supply and economic stability. However, intensive agricultural practices can also result in significant environmental impacts [22], including the generation of waste, greenhouse gas emissions, and the degradation of air, water, and soil quality when residues are not properly managed [23].

The agricultural sector can promote technologies, generate jobs, and improve income, but it faces the challenge of rural poverty, which represents 80% of poor people worldwide. The fostering of agricultural innovation through co-funding, knowledge centers, support services, partnerships, collaborative institutions, and funding for farmers promotes solutions to improve rural income and sustainability [24]. Those contributions are in accordance with the Sustainable Development Goals (SDG) 01, 02, 09, 12 and 17 from the 2030 Agenda [25]. Valorization of agriwaste is a path to promote extra income for rural people, sustainability, and circular economy [26]. In this section, we will prioritize crops and correspondent bio residues with potential use as inputs for the textile industry.

Sugarcane (*Saccharum officinarum* L.) presents the biggest global cultivar production in volume, achieving more than 2 billion tons annually [16]. It is the main raw material for sugar and alcohol production. The bagasse is the residue outcome from these processes, accounting for 30% of sugarcane plant residues. Such residue is mainly exploited for biofuel, paper, textile, and feed industries and less for cellulose and nanocellulose recovery [27]. Discarded sugarcane waste can contaminate groundwater and soil, altering their chemical and physical condition [28].

Maize (*Zea mays* L.), also known as corn, represents 12% of the crop volume worldwide, counting 1.2 billion tons in 2023 [29]. The grain produces food, starch, biofuels (mainly ethanol), animal feed and pharmaceuticals. Leaves, husks, and cobs are the major agriwastes of corn. Husks comprise 15% of the corn waste volume and are currently destined for landfill or incinerated after harvest. To optimize this residue management and reduce carbon emissions, corn husks could be exploited as fiber sources within an upcycling proposal [30].

Rice (*Oryza sativa* L.) crop is the agricultural practice with the biggest rate of carbon emissions (1.0 kg CO_2_ eq/kg), higher than all other cereal rates combined. In 2023, rice producers harvested 800 million tons of grains [29], representing 9% of the world’s crops [21]. Rice wastes, i.e., husks, straw, and bran, are potential sources of fibers, silica, and anthocyanins for industry [31,32].

**Figure 1:**
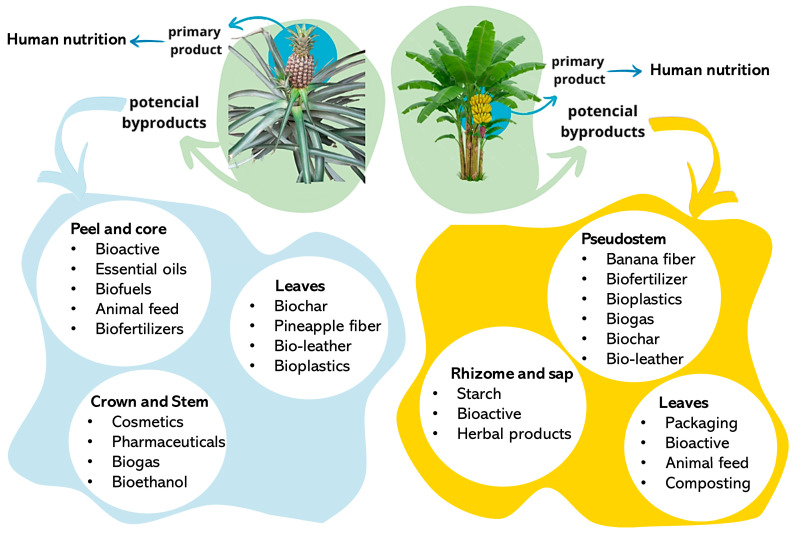
High-volume fruit crops that generate large amounts of agro-industrial residues after single harvests (e.g., banana, pineapple, coconut). These residues are rich in lignocellulosic components and other valuable compounds that can be upcycled into textile and bio-based materials.

Fruit crops significantly grew in past years, achieving 952 million tons in 2022 [16]. In 2023, banana was the fruit with the most expressive production and growth, accounting for 139 million tons [29]. The entire banana tree structure (stem, leaves, roots and heart) instantly becomes waste biomass after one single fruit harvest (Figure 1).

The voluminous waste described above, usually disposed of, can attend many purposes, such as fertilizers, animal feed, biofuel, bioenergy, biopolymers, nutraceutical products, and textile yarn [22]. Similarly, one single pineapple harvest results in the disposal of 25 thousand plants per hectare that are usually incinerated (Figure 1). The pineapple leaves can reach up to 2 m long and are composed of fibers that, after recovered and treated, can be commercialized as a fine-quality yarn named Pinatex [33].

## 4. Upcycling of Agro-Industrial Waste into Textile Processing

**Figure 2:**
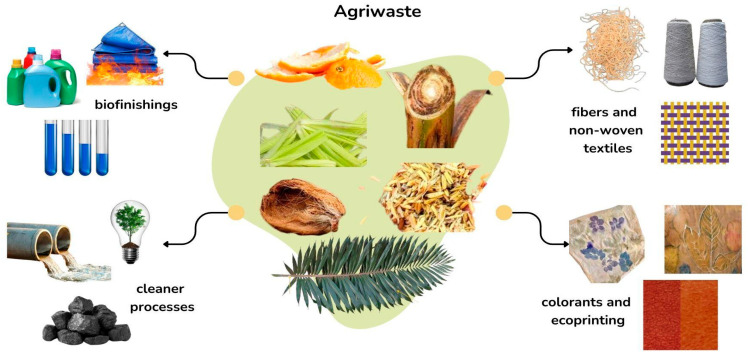
Proposals for the conversion of vegetable-based agricultural residues into inputs for textile manufacturing.

Field agrowaste or agriwaste consists of leaves, rinds, husks, peels, stems, and plant matter, including flowers. Such residues hold a significant potential for upcycling into key input to the textile industry. From fibers to finishing agents, repurposing such materials can enhance the value chain, contribute to a more sustainable production system and redirect material that otherwise would be discarded or underutilized. Fiber extraction, obtainment of finishing agents and pigment extraction are important upcycling processes that involve vegetable agriwaste to generate inputs for the textile industry (Figure 2). Several agro-industrial residues—including banana, coconut, and pineapple—serve as exemplary sources of biomass that can be transformed into fibers, pigments, finishing agents, or biocomposites through different upcycling processes, as detailed in the following subsections.

### 4.1. Textile Fibers from Tucum Palm Tree: A Study Case

Recently, our research group extracted, characterized, bleached and dyed fibers from a common palm tree found in South Brazil, known as *tucunzeiro* [9]. Tucum (*Bactris setosa* Mart.), a native palm tree from Atlantic forest provides a fruit as its primary product. Adaptable to humid or dry climates, this type of palm tree can grow in tropical and subtropical biomes worldwide [34]. Traditionally consumed by South American natives in their culinary, folk medicine and handcrafts, tucum’s technological and biotechnological potentials remain underexplored [35].

After the fruit harvest, leaves, stem, rachis and thorns become residues. Tucum leaves are composed of leaflets that can be manipulated, one by one, for fiber extraction through a manual and ancient technique. Although slow, manual extraction is efficient, clean and does not harm the fibers maintaining its properties. Currently, it is the most appropriate extraction process for these fibers [9].

The recovered tucum fibers were 10 to 39 cm long, being an above-average fiber length when compared to most common natural fibers. This length variation is due to the elliptical shape of the leaves [9]. The FTIR spectrum confirmed the characteristic cellulose structure of tucum fibers, showing a strong O–H stretching band that indicates the presence of abundant hydroxyl groups, responsible for the polar and hydrophilic nature of lignocellulosic materials. Its degradation was similar to other lignocellulosic fibers as verified by thermogravimetric analysis. With initial elastic reaction when strained, tucum fibers endured through added stress until rupture revealing a higher tensile strength (942 MPa) than cotton or sisal. This strength could be related to the presence of phytoliths on fibers surface that appeared in SEM analysis [9]. Phytoliths are a natural coating that improves fiber strength and function as a barrier to its degradation making it more suitable for spinning and developing mixed composition yarns. SEM analysis also revealed considerable fineness of tucum fibers, an average of 28.3 µm, measurement lower than that of human hair.

The fibers underwent pre-treatment processes such as bleaching to homogenize its surface, whitening its natural color and softening the material. Pre-finishing processes add value and prepare natural fibers to sequential finishing such as dyeing and printing [36]. Tucum fibers underwent bleaching and dyeing processes using synthetic inputs, to test its viability for currently used industrial processes. The tucum fibers were bleached with hydrogen peroxide, transforming irregular green fibers into homogeneous and milky color ones [9]. After this pre-bleaching process, the tucum fibers were dyed with an aqueous solution of Pink Tricel NG-LRB direct colorant at 100 °C for 40 min, in a comparison study with cotton fabric and viscose fabric. The fibers presented a superior homogeneous darker color shade with no stains when compared to the fabrics, showing a higher absorption dye yield [9]. Further quantitative data on the chemical composition and bleaching yield of tucum fibers, expressed relative to the weight of the original leaf material, can be found in the detailed experimental study by Flohr et al. (2024) [9].

Tucum is a versatile crop, underexplored, with good primary products and byproducts. Its fibers exhibit appropriate properties as its fineness, high strength, hydrophilicity and long length, suitable for producing good quality yarn for final applications such as apparel industry or fabrication of composite material. It also indicates compatibility with bleaching, finishing and spinning. These features place tucum fibers as an environmentally friendly alternative to traditional textile fibers.

### 4.2. Fiber Extraction

Many commercial textile fibers are either plastic-based or derived from natural sources, which require significant resource consumption. The pursuit of more sustainable alternatives has driven extensive research into the valorization of plant residues as sources of natural or mixed yarns, and as reinforcements for biocomposites (Table 1).

Among tropical crops, banana (*Musa* spp.) [22] and pineapple (*Ananas comosus*) [33] have emerged as promising raw materials. Banana stems and leaves yield fibers of varying thickness: inner stem fibers can be spun into fine, silk-like yarns, while outer stem fibers are coarser and suited for products such as ropes and crafts [30,37]. Similarly, pineapple leaf fibers, when properly retted and bleached, yield resistant, bright, and hydrophilic yarns known as pinatex [33], possessing desirable mechanical and aesthetic properties for textile applications.

Other agricultural residues, such as corn husks [45], barley straw [43], areca leaves [44], and okra stems [26], also produce lignocellulosic fibers compatible with spinning, blending, and composite manufacturing. These fibers often exhibit good tensile strength, hydrophilicity, and flexibility, depending on the retting and treatment process employed. For instance, enzyme-retted corn husk fibers are pliable and strong; barley straw fibers possess mechanical properties comparable to cotton; and okra fibers, extracted via water or chemical retting, can be combined with jute in non-woven structures.

Scientists have developed novel biotextiles from plant-based residues beyond conventional spinning [48]. Fermented fruit leaves, combined with bacterial cellulose, yield leather-like materials suitable for shoes and bags, while kombucha tea and sugarcane residues can generate biodegradable, short-lived fabrics [49]. Research groups have engineered mango-derived films into tensile-resistant biotextiles, with edible or extended-use variants determined by the surface finish applied [47]. Collectively, these advances demonstrate the potential of agricultural residues to serve as renewable, functional materials in the textile sector.

### 4.3. Obtainment of Finishing Agents

Textile finishing imparts essential commercial characteristics to fabrics, such as texture, color, softness, and functional performance. However, it remains one of the most environmentally damaging steps within the textile chain. Conventional finishing processes consume large volumes of water and involve a variety of chemical agents—including heavy metals, formaldehyde, surfactants, and bleaching compounds—many of which are toxic and persistent, posing risks to human health and ecosystems [36,50].

In the context of the transition toward a circular bioeconomy, a study conducted by researchers using mandarin peel waste (*Citrus reticulata* L.) demonstrated the biotechnological potential of this residue to replace conventional chemical finishing processes in textiles. The authors investigated the fermentation of mandarin peels by a bacterial strain of *Pseudomonas* sp. HRJ16, aiming to produce a laccase-type enzyme with high catalytic activity and stability under extreme conditions [36]. The production of the bacterial exudate (HRJ16 laccase) was optimized using Response Surface Methodology (RSM), employing low-cost agro-industrial waste as a fermentation substrate. After purification, the enzyme was characterized by its biochemical and catalytic properties, revealing high tolerance to variations in temperature, pH, salts, cations, and surfactants, maintaining approximately 80% residual activity even under adverse conditions. These characteristics made HRJ16 laccase particularly suitable for textile applications, where it is used in bioscouring (removal of natural fiber impurities), biobleaching, and synthetic dye decolorization. The enzymatic treatment applied to denim fabrics yielded remarkable results, achieving complete bioscouring in approximately six hours and spontaneous decolorization of the resulting effluent, indicating the enzyme’s dual function as both a cleaning agent and a wastewater treatment catalyst [36].

To replace halogenated compounds and toxic solvents traditionally used in textile finishing [36,50], researchers developed an eco-friendly flame-retardant coating composed exclusively of natural-origin materials. Inspired by the classical dye-fixation process, the study developed a biomass-based coating system consisting of tannin (TA), tartar emetic (TE), and ferrous ions (Fe^2+^), applied to cotton fabrics without the use of phosphorus, bromine, chlorine, or hazardous organic solvents. In this system, tannin—a phenolic compound derived from plant biomass—acted as a charring agent, fixed onto the cotton fiber surface by tartar emetic, which functioned as a binding agent similar to the dye-fixation mechanism in aqueous medium. Subsequently, Fe^2+^ ions coordinated with the hydroxyl groups of the TA–TE complex, catalyzing the formation of graphitized, thermally stable carbon residues, responsible for the high flame resistance. The experimental results demonstrated excellent flame-retardant performance and remarkable durability of the coating. Even after 100 washing or friction cycles, the limiting oxygen index (LOI) remained around 27%, indicating consistent non-flammability. The treated fabrics also passed horizontal flammability tests, exhibiting minimal flame spread rate [36,50].

In search of sustainable alternatives for developing functional textile finishes, researchers proposed using silica nanoparticles extracted from rice husk [51]—an abundant agro-industrial residue—to impart water repellency to eri silk fabrics. The process involved the extraction of silica nanoparticles and their subsequent application onto the fabric, aiming to modify its surface properties without compromising structural or aesthetic characteristics. The water-repellent efficiency was evaluated through contact angle, absorption, and spray tests, essential parameters for characterizing the hydrophobic behavior of textile surfaces. Furthermore, a silicone-based polymer was applied to enhance repellency, resulting in a hybrid coating with superhydrophobic properties. Characterizations by Scanning Electron Microscopy (SEM), Fourier Transform Infrared Spectroscopy (FTIR), and Energy-Dispersive X-ray Spectroscopy (EDX) confirmed the uniform presence of silica nanoparticles on the eri silk fibers and the formation of a continuous surface layer responsible for the water-repellent effect [31].

Lyocell, a regenerated cellulose fiber widely recognized for its sustainable origin and biodegradability, nevertheless presents a major limitation—high flammability, which restricts its use in applications requiring greater thermal safety. To overcome this challenge, researchers developed a biomass-based flame retardant derived from vitamin C (VCFR), designed and synthesized as an eco-friendly alternative to halogenated and phosphorus-based additives [52]. The finishing treatment was carried out through esterification, promoting the chemical bonding of VCFR to the lyocell fiber surface, yielding flame-retardant lyocell (FR-lyocell) fabrics. Thermal performance tests revealed excellent flame-retardant efficiency and durability. The LOI value of the FR-lyocell reached 39.8%, remaining at 26.4% even after 30 washing cycles, demonstrating strong chemical stability and washing resistance. Cone calorimeter tests further confirmed the protective effect of the coating, showing an 89.5% reduction in the peak heat release rate and a 54.5% reduction in total heat release compared with untreated fabric [52].

Making leather more biodegradable and environmentally compatible at the end of its life cycle has become a research priority in sustainable leather processing. In this context, the study proposed an innovative and eco-friendly tanning system utilizing sugarcane bagasse (*Saccharum officinarum* L.) an abundant byproduct of the sugar and alcohol industry—as an alternative tanning agent. The process involved converting cellulose and hemicellulose from bagasse into dialdehyde polysaccharides (DAPB) through controlled oxidation, producing compounds capable of chemically crosslinking with collagen in animal hides [53]. After the conventional pre-tanning steps (soaking, liming, and deliming), goat skins were tanned with the DAPB obtained from hydrolyzed bagasse. The resulting leather showed resistance to enzymatic degradation by collagenase, indicating the tanning efficiency of the dialdehyde polysaccharides [50]. Moreover, the tanned leathers exhibited mechanical properties comparable to those of chrome-tanned leathers, maintaining suitable strength and flexibility for commercial applications [53].

This study, developed by our research group, aimed to create a sustainable flame-retardant treatment for cotton fabrics, using a hybrid organic–inorganic coating composed of chitosan, phytic acid, APTES (3-aminopropyltriethoxysilane), and eggshell powder, applied at concentrations of 2% and 4%, in one or two coating cycles [54]. This formulation seeks to replace halogenated and phosphorus-based compounds with natural and agro-industrial waste materials, combining technical performance with environmental sustainability. Fourier-transform infrared spectroscopy (FTIR) confirmed the formation and deposition of the hybrid layer on cotton fibers through the appearance of characteristic organic–inorganic bonding bands. Thermogravimetric analysis (TGA/dTGA) revealed a notable increase in thermal stability, characterized by a higher char yield and a shift in the main cellulose degradation peak, indicating an effective protective action under heat exposure. Vertical flammability tests demonstrated that all coated samples achieved self-extinguishing behavior within 12 s, meeting NFPA 701 standards [55]. Among the tested formulations, the sample containing 2% eggshell powder with two applications (S2%–II) exhibited the best balance between flame retardancy and mechanical performance. Tensile strength tests indicated improved fiber cohesion after treatment, while SEM micrographs confirmed uniform coating deposition and particle integration into the fiber matrix. Colorimetric analysis revealed that the treatment did not significantly alter the natural color of cotton, preserving its original appearance. Although washing resistance remains a limitation due to the biopolymeric nature of the components, the treated samples remained stable over time, with no microbial growth or staining, demonstrating potential for use in upholstery and covering fabrics that are not subjected to frequent washing. The results confirm the technical and environmental feasibility of using agro-industrial residues, such as eggshell waste, in functional finishing formulations, reinforcing the potential for developing eco-efficient, low-impact textile coatings. This innovative approach strengthens our research group’s commitment to waste valorization, the principles of the circular economy, and sustainability within the textile sector.

### 4.4. Colorant Extraction

Bioresidues are also destined to provide added value as colorants on processes of textile printing and dyeing. Natural dyes and pigments can be extracted from different parts of plants, such as roots, branches, leaves, fruits and seeds, in order to decrease the use of synthetic dyes, which cause water, air and soil pollution [56]. Their color features can vary according to the chemical class of the constituent molecules, such as anthocyanins, carotenoids, anthraquinone, flavones, and indigoids. After dyeing, the color fixation of natural colorants in textile substrates can be a challenge. To maintain the color and obtain darker shades, fixation agents known as mordants are used. The most popular mordants are salt, metal extracts, and acids; however, plant-based agents named biomordants have been studied [57]. In the past decade, groups worldwide have performed the recovery of colorant molecules from vegetable residues and their subsequent application in textile dyeing (Table 2).

The ecoprinting technique is a non-patterned print, handmade process, related to slow production with added value. It is a clean process because it does not require toxic agents and does not generate contaminated wastewater. Using many types of flowers or leaves, ecoprint achieves better fixation with biomordants or alums than heavy metal-based mordants. Although simple, low cost, and applicable, the quantity of papers means that it is still scarcely used [58,59].

Due to its features, natural dyes present some fixation issues and do not always result in intense and/or stable shade colors. Beetroot pigment is an example of this issue. Therefore, studies have been testing fixation agents or innovative ways to hold this bethalayne pigment in textiles. Functionalizing protein fabrics with acetic acid as a pre-treatment before dyeing improves the process of fastening and results in a better fixation of color even after washing [68]. In other specific cases of pigment origin, fixation agents are not applied. Due to its inherent properties and chemical composition, wood liquid residue or bark colorants do not always require mordants to achieve a proper dye. Therefore, it consumes less water and energy than regular dyeing processes [65,66].

### 4.5. Agro-Industrial Residues and Plant-Based Resources for the Alternative Leathers Manufacturing

The search for sustainable alternatives to animal leather has led to the development of bio-based and vegan leathers derived from agro-industrial residues and renewable plant biomass. These materials represent technological responses to the environmental and ethical challenges of the conventional leather industry, which involves high water consumption, toxic tanning agents (chromium salts, aldehydes), and significant greenhouse gas (GHG) emissions associated with livestock and chemical processing [69].

Unlike petroleum-based synthetic leathers, plant-derived leather substitutes can be produced from renewable biomasses rich in polysaccharides, lignin, and proteins, often originating from agricultural waste streams. Their formation relies on the extraction and crosslinking of natural macromolecules—such as cellulose, hemicellulose, lignin, pectin, and starch—that, when plasticized or blended with bio-polymers, form cohesive and flexible films suitable for coating or lamination. A well-known example is cactus leather, developed in Mexico and marketed under the name Desserto^®^. It is produced from the leaves of *Opuntia ficus-indica* L. cactus through dehydration, mechanical pulping, and polymer blending, without cutting the plant. The process exploits the pectin and cellulose content of cactus tissue, combined with natural oils, to create a durable and flexible biopolymer network. Cactus cultivation requires minimal irrigation and is well adapted to arid regions, making it consistent with circular economy and water-sustainability principles [70].

Aiming to develop sustainable and ethical alternatives to conventional leather, researchers proposed the production of plant-based (vegan) leather using fungal biomass grown on bread waste. The process employed a bubble column bioreactor, where nutrients extracted from discarded bread served as a substrate for fungal cultivation, demonstrating the potential of agri-food residue valorization for generating high-value bioproducts. The resulting fungal biomass underwent vegetable tanning using different natural tannins—tara, myrobalan, chestnut, and Indusol ATO—followed by a mild alkaline treatment to isolate the fibrous cell wall material. Subsequently, the researchers prepared multilayer composite sheets by combining the tanned biomass with the fibrous material, which were then post-treated with glycerol and a bio-based binder to enhance flexibility and mechanical strength. Among the tested formulations, the myrobalan-tanned composite exhibited the highest flexibility (14.8% elongation at break), while the tara-tanned composite showed the greatest tensile strength (20.5 MPa). Scanning electron microscopy (SEM) analyses revealed smoother and more uniform surface morphologies in chestnut- and Indusol ATO-tanned composites after post-treatment, resembling the texture of natural leather. Overall, the results demonstrate that multilayer fungal biocomposites derived from agri-food waste are a promising and eco-friendly alternative to animal leather, effectively combining organic waste recycling, low environmental impact, and mechanical properties comparable to commercial materials [71].

In an effort to develop sustainable alternatives to traditional leather, researchers produced a semi-synthetic leather using fruit and vegetable waste as a key raw material, combined with acrylic polymer resin and functional additives. The formulation included glycerol and polyvinyl acetate (PVA) to enhance flexibility and resistance to cracking, essential characteristics for materials intended for footwear and upholstery applications. The semi-synthetic leather film was obtained by casting a homogeneous mixture of fruit and vegetable waste powder, acrylic resin, and additives into an aluminum mold, followed by drying at 120 °C for 4 h. The study systematically optimized the concentrations of each component—acrylic resin, PVA, glycerol, and waste powder—to identify the composition that provided the best mechanical performance and structural integrity. Mechanical and structural characterization revealed that the optimized formulation (40% acrylic resin, 5% fruit–vegetable waste powder, 5% PVA, 5% glycerol, and 45% water) achieved tensile strength of 112.1 N, elongation at break of 91%, tear strength of 15.06 N, and flexural rigidity of 62.44 mg·cm^2^. These properties were strongly correlated with the proportion of acrylic resin and waste powder in the blend. Furthermore, the resulting material exhibited excellent abrasion resistance, withstanding 20,000 abrasion cycles without significant surface degradation [72].

The areca leaf sheath, once discarded as agricultural waste, has recently gained prominence as a renewable raw material for sustainable product manufacturing. With the continuous growth of arecanut cultivation, large volumes of leaf sheaths are produced annually. However, traditional small-scale industries focused on disposable tableware cannot fully utilize this abundance. This surplus has encouraged entrepreneurs to explore innovative, high-value applications, such as the development of palm leather, a biodegradable and pollution-free alternative to conventional animal leather. The study highlights a successful start-up in Shivamogga, Karnataka (India), established under the Start-up India initiative, which converts areca leaf sheath waste into palm leather products, including indoor slippers, diary covers, and vanity bags. The production process is environmentally friendly, relying on natural processing methods that eliminate chemical tanning and minimize waste generation, exemplifying the conversion of agro-waste into value-added materials. Economic assessment confirmed the strong financial feasibility of the enterprise. Arecanut farmers earned additional income of approximately ₹38,000 (≈USD 455) from the sale of leaf sheaths, while the entrepreneur achieved net profits of about ₹116.22 (≈USD 1.40) per pair of slippers, ₹76.98 (≈USD 0.92) per diary cover, and ₹253.52 (≈USD 3.00) per vanity bag. The project demonstrated a net present worth (NPW) of ₹7.2 crores (≈USD 860,000), a benefit–cost ratio (B:C) of 1.49, and an internal rate of return (IRR) of 134%, all of which indicate excellent economic viability and market potential [73].

Researchers investigated strategies for the management and valorization of agro-food waste, focusing on green kiwi peel (GKP)—a major byproduct of kiwi consumption—as a renewable raw material for the development of bio-based polymer films. Due to its natural composition rich in cellulose, hemicellulose, and pectin, GKP represents a promising substrate for circular biomanufacturing processes aimed at achieving a zero-waste economy. In the study, the researchers sought to fully valorize GKP by combining material functionalization and biotechnological processing. The kiwi peels were first mechanically ground and then treated either with citric acid or with commercial enzymatic preparations to produce biodegradable films. The enzymatic treatment selectively consumed certain biopolymer components, altering the ratio of cellulose, hemicellulose, and pectin, which enabled tuning the mechanical properties of the resulting films according to different processing conditions and performance requirements. The researchers also demonstrated that the acidic liquid fraction generated as a byproduct of enzymatic hydrolysis—rich in glucose and fructose—could be repurposed as a nutrient medium for cultivating industrially relevant yeast strains, including *Saccharomyces cerevisiae*, *Yarrowia lipolytica*, and *Rhodotorula toruloides*. This secondary application highlights the potential for integrated resource utilization, where all fractions of the agro-waste are converted into valuable products [74].

Researchers investigated the reuse of orange peel waste as a raw material for vegan leather production, promoting the sustainable utilization of agri-food residues in the manufacture of environmentally friendly materials. Different citrus peel formulations were evaluated, incorporating pomelo-based reinforcing agents to enhance the mechanical strength and flexibility of the resulting biocomposites. The researchers found that the composite produced from grapefruit peel reinforced with pomelo exhibited the best overall performance. The material achieved a tensile strength of approximately 472 N/cm^2^, elongation at break of 67.28%, and tear resistance around 14.28 N/cm^2^, values superior to those obtained from sweet orange peel composites with similar reinforcement. These results indicate that the addition of natural reinforcements significantly improves the cohesion and elasticity of the material, bringing its properties closer to those of commercial synthetic leathers. The researchers concluded that recycling citrus peel residues, such as orange and grapefruit peels, shows great potential for developing functional, durable, and flexible vegan leathers. This approach represents a low-cost and biodegradable alternative to conventional leather, while also offering a sustainable solution for managing citrus industry waste, contributing to the advancement of the circular economy and innovation in bio-based materials [75].

Other representative cases include (I) Pineapple leather (Piñatex^®^): produced from pineapple leaf fibers, an abundant residue after fruit harvest. The fibers, rich in cellulose and lignin, are mechanically separated, purified, and dispersed in a polylactic acid (PLA) or polyurethane matrix, forming a non-woven composite structure with good tensile strength and flexibility; (II) Apple leather: derived from pomace residues from juice and applesauce industries. Its pectin and starch fractions are combined with natural latex or corn starch biopolymers, yielding biodegradable sheets with high elasticity and low water permeability; (III) Grape leather (Vegea^®^): obtained from grape marc, composed of skins, stalks, and seeds remaining after wine production. These residues are processed through solvent-free extraction and esterification, producing films rich in polyphenols and lignin derivatives that enhance UV stability and aesthetic quality [76].

The valorization of such agro-industrial by-products demonstrates how biochemical and mechanical routes can transform cellulose- and pectin-rich residues into flexible biomaterials suitable for fashion, upholstery, and automotive applications. These materials combine the structural reinforcement of lignocellulosic fibers with polymeric matrices derived from renewable resources, achieving mechanical strength and durability while maintaining biodegradability. Furthermore, research has investigated the incorporation of plant proteins (soy, zein, gluten) and lignin or tannin extracts as natural crosslinkers or plasticizers, improving cohesion and resistance. These compounds, extracted through alkaline hydrolysis or solvent extraction, react with hydroxyl or carboxyl groups in the polysaccharide matrix, enhancing film formation and mechanical performance. The inclusion of nano-cellulose or biochar particles from agricultural residues can further reinforce the composite, increasing tensile strength and dimensional stability.

Despite the progress achieved, challenges remain in scaling up production, ensuring uniform mechanical behavior, and achieving cost parity with synthetic or animal leather. Future directions include green-solvent processing, enzymatic crosslinking to replace petrochemical additives, and the integration of by-product valorization chains (e.g., pineapple, banana, coconut, or grape residues) within regional bioeconomy systems [69].

### 4.6. Other Upcycling Options for the Textile Industry

Agriwaste materials can also be exploited as solutions for the textile industry, enhancing sustainability when applied to general industrial processes such as bioenergy generation and adsorption of dyes and pollutants from wastewater.

Biomass can be defined as every natural residue that is appropriate to produce cleaner energy through carbon combustion [43]. Regarding agriwaste, banana peel, and other banana tree residues [22], barley residues [43], sugar cane bagasse [53], cotton ginning waste [77], teff grain straw, coffee husk, corn cob, and sorghum stalk [78], are considered suitable biomass for energy production either by combustion, pyrolysis or gasification.

Biochar consists of biomass transformed thermally into a byproduct rich in carbon composition. This byproduct is suitable as a fertilizer agent for agricultural purposes, pollutant absorbent, or subtracts for bioenergy production. Agricultural residues are one of the preferred biomaterials for producing biochar. Biomass was produced by recovering lignocellulose from sugarcane bagasse, rice straw, guava, custard apple (*Annona squamosa* L.), macadamia husk, and pistachio husk. The lignocellulosic biomass combined with textile sludge underwent a microwave-assisted wet torrefaction process, resulting in a greener biochar. It displays more energy efficiency and 57.5% less gas emissions than bituminous coal [79].

Wastewater is a challenge to achieving a cleaner textile industry production. It is usually discarded carrying dyes, oils and other chemicals, even after treatment. The search for better effluent treatments is a long-due pursuit. Regarding colorant degradation or sorption, it has been verified that onion peel is an efficient asset in decoloring wastewater [56] and that fibers extracted from poplar seeds have better oil sorption than cotton fibers [80]. Accordingly, cassava waste converted into a biochar adsorbent through milling and hydrothermal carbonization treatment could efficiently adsorb dyes [81] and rice husk biochar obtained by drying and combustion resulted in a compatible dye and heavy metal adsorbent to diminish pollution from wastewater [82]. Vasconcelos et al. (2025) identified multilayer absorbency properties in *Astrocaryum aculeatum* unaltered seeds being capable of removing up to 50 mg/g of colorant and from textile wastewater in linearly influenced pH without previous treatment [83].

## 5. Economic Aspects and Sustainability in the Utilization of Agro-Industrial Waste

### 5.1. Economic and Technical Feasibility Analysis

The economic and technical feasibility analysis of the reuse of agro-industrial waste in the manufacturing industry presents itself as an innovative and sustainable solution, contributing to reducing environmental impacts and generating added value [84]. This practice aligns with the principles of the circular economy, aiming at transforming waste into resources, minimizing waste, and promoting efficiency in the production process [85].

Analyzing economic feasibility involves assessing the costs of reusing waste, categorized into three main components: collection and transportation, processing technology, and adaptation of industrial processes [86]. Planning the logistics for collecting agro-industrial waste is one of the first steps to consider. Dispersed rural locations often increase transportation costs [87]. In addition, it is necessary to invest in infrastructure for adequate storage, ensuring that the waste remains in appropriate condition for later processing [88]. Reusing waste requires using specific technologies for sorting, cleaning, and transformation into usable materials. Extruders, fiber extraction machines, and effluent treatment systems represent significant initial investments [5].

However, the cost of this equipment can be diluted over time, considering the reduction in the use of raw materials and the added value to the products. Companies that wish to incorporate waste into their production chains must adapt their industrial processes, which can involve everything from adjusting existing equipment to training their workers [89]. Despite the high initial cost, these modifications can result in greater efficiency and reduced waste in the long term. The economic benefits associated with the reuse of agro-industrial waste are diverse and range from reducing operating costs to generating new sources of revenue [90].

Using waste as inputs replaces the dependence on conventional raw materials, which are generally expensive and fluctuate according to the market. For example, plant fibers extracted from waste, such as tucum, can replace conventional fibers in textile applications, reducing total production costs [9]. Agro-industrial waste transforms into innovative, high-demand products such as sustainable fabrics, natural auxiliary chemicals, softeners, flame retardants, and biochar [88]. These products can conquer market niches that value sustainability, especially in developed countries. As environmental awareness grows, consumers and businesses are prioritizing sustainably manufactured products. The increased demand for sustainable goods opens export opportunities and business partnerships in markets that impose restrictions on non-sustainable products. In addition, governments often offer tax incentives, low-interest financing, and subsidies to companies that adopt circular economy practices, significantly reducing implementation and operating costs [91].

The technical feasibility of reusing agro-industrial waste depends directly on the quality of the products obtained and the efficiency of the processes involved [92]. Studies show that materials from agro-industrial waste, such as plant fibers and natural dyes, have technical properties compatible with conventional products [93]. For example, fibers extracted from tucum have mechanical properties suitable for textile applications, while natural dyes offer good color stability when processed correctly [9].

Clean technologies, such as effluent treatment and biochar production, have significantly reduced the carbon footprint associated with waste reuse [94]. In addition, generating energy from organic waste is a promising byproduct of these processes. Many of the techniques already used in industry can be adapted to incorporate waste. For example, ecoprint, a botanical printing technique, allows the use of leaves, flowers, and other plant waste to create unique fabric patterns [59]. Investments in research and development are essential to overcome technical barriers, such as the uniformity of the resulting products and the energy efficiency of the processes. Promising areas include the optimization of extraction techniques and the creation of new materials from waste. A relevant example is the study of plant fibers extracted from unconventional plants, which have proven to be a viable alternative to synthetic fibers in terms of cost and performance [95]. Another scenario is the use of fruit peels as a source of natural dyes for dyeing fabrics, generating ecological products with high added value [96].

The analysis of the economic and technical feasibility of reusing agro-industrial waste shows that this practice is both a necessity and an opportunity for the manufacturing industry. Despite the initial challenges, such as high implementation costs and technological barriers, the economic and environmental benefits are significant. With investments in research, innovation, and incentive public policies, it is possible to integrate agro-industrial waste into production chains efficiently and profitably, contributing to global sustainability and promoting the transition to a circular economy model. Table 3 shows the possible sources of costs and each category’s technical feasibility.

### 5.2. Sustainability and Circular Economy of Agro-Industrial Waste in the Textile Industry

The inadequate disposal of agricultural waste leads to the anaerobic decomposition of organic matter, releasing large amounts of GHGs such as CO_2_, CH_4_, and N_2_O. This not only accelerates climate change but also undermines global sustainability efforts [104]. Agro-industrial waste further exacerbates this issue by generating pollution across soil, water, and air, contributing to energy waste and biodiversity loss throughout the food supply chain [105].

Economically, the ineffective management of agricultural residues results in the underutilization of valuable raw materials, increasing dependence on landfills and incineration, and environmentally damaging through unsustainable practices. Conventional reuse methods, such as composting, animal feed, or bioenergy, often lack scalability or profitability [106].

At the same time, the textile and clothing sector remains one of the most environmentally inefficient industries. It is energy-intensive and heavily reliant on thermal processes, generating significant GHG emissions [107,108]. Moreover, its water usage is excessive, particularly in dyeing and finishing. Up to 15% of the dyes used are lost and discharged into wastewater, creating costly treatment demands due to their persistence and toxicity [109,110].

Solid waste generation throughout the textile supply chain, including offcuts, packaging, and unsold goods results in high disposal costs. Most of this waste ends up in landfills due to the use of non-recyclable materials [107]. The widespread use of synthetic fibers derived from petrochemicals not only increases emissions but also contributes to microplastic pollution, especially through post-consumer waste [111,112].

The global resource extraction rate of 106.6 billion tons in 2023 reflects an unsustainable and economically risky linear “extract-produce-dispose” economy [113]. This model fails to incorporate circular strategies, limiting material availability and resilience, requiring the development of circular models to promote efficiency, reduce production costs, and enable poverty reduction through value generation [114].

Within this context, SDG 12, Responsible Consumption and Production, promoted by the UN, advocates the 3Rs (reduce, reuse, recycle) and supports sustainable industrial practices. Circular economic strategies in the textile sector emphasize material reuse, waste minimization, and value recovery from agro-industrial residues [115]. Valorization, a key principle of circularity, focuses on converting waste into high-value products, improving cost-effectiveness, and reducing environmental liabilities [116].

Biobased solutions, including plant-based polymers and fibrous residues, offer scalable alternatives for manufacturing eco-efficient textiles. The rising demand for agricultural production further amplifies the urgency of managing its by-products. Reusing these residues lowers emissions and introduces low-cost inputs into industrial supply chains [117]. In this scenario, raw materials from renewable and recycled sources are increasingly adopted to mitigate material depletion and reduce production costs. Integrating sustainable design with bioeconomy principles can enhance product lifecycles and process resilience [118].

The upcycling of agri-food waste into value-added products through physical, chemical, or enzymatic processing represents a viable path for eliminating landfill accumulation while creating economic returns [114,119]. Agro-industrial residues such as fibers, polysaccharides, and enzymes can be transformed into low-cost raw materials for the chemical and textile sectors [120]. Nonetheless, challenges remain regarding raw material heterogeneity, logistics, and process optimization. Variations in moisture, contaminants, and composition affect the consistency and profitability of industrial applications. Pre-treatment steps such as sorting and drying are essential to ensure standardized inputs and cost-effective operations [106,121].

Plant-based fibers, e.g., from bananas, pineapples, bamboo, and corn demonstrate a high potential for converting waste into economically valuable textile inputs [122]. Promoting sustainability in this sector is more than an environmental imperative, it is an economic opportunity. A circular approach can transform the textile industry from a pollution-intensive system into a driver of innovation, job creation, and responsible resource use [123].

Despite the numerous claims regarding sustainability benefits, the literature lacks quantitative analyses that validate the actual environmental life cycle impact of these new processes. The present review search revealed only one comprehensive review article, by Phan et al. (2021), which conducted a life cycle assessment (LCA) comparing natural dyes extracted from agro-food waste with a synthetic anthraquinone dye [11]. The results of this study indicated that environmental viability is highly dependent on the dye class and the processing route. Anthocyanin dyes (from grape pomace) and quinones (from walnut husks), when applied as a crude extract, proved to be environmentally competitive or even superior to their synthetic counterparts. In contrast, the extraction of carotenoids (from tomato pomace) showed a significantly higher environmental impact due to the intensive use of organic solvents. The study also identified the biomass drying stage as a critical hotspot, accounting for a considerable portion of the total environmental impact in several scenarios. Their conclusion was that using the crude extract was the most ecological approach in all cases and indicated that the purification and concentration processes could impair the sustainability gains.

Therefore, the scarcity of studies like this highlights a critical gap, making it imperative to conduct more LCA and techno-economic analysis (TEA) studies to ground strategic decisions and ensure that the transition to bio-based inputs truly represents an advancement for industrial sustainability.

**Figure 3:**
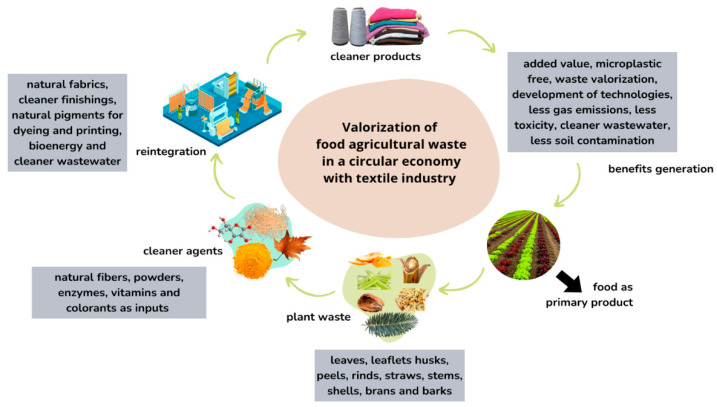
Proposal of a textile upcycling model that reintegrates vegetable wastes into the production chain.

The current linear flow of extraction–production–disposal must shift to a circular model where agro-industrial waste is reintegrated into the production chain [124]. As illustrated in Figure 3, this circular flow includes biomass harvesting, processing, waste separation, its transformation into value-added materials, reintegration into manufacturing, and the generation of socio-environmental benefits, thus closing the loop in a sustainable and resilient manner.

## 6. Conclusions and Future Perspectives

This review provides a critical assessment of the potential of plant-based agro-industrial residues as renewable feedstocks for a more sustainable textile industry. The upcycling of these materials demonstrates substantial environmental and economic advantages by promoting a resource-efficient production model that reduces landfill accumulation and dependence on virgin raw materials.

However, translating this concept to an industrial scale still faces significant scientific and operational challenges. The intrinsic heterogeneity of agro-based residues, coupled with the lack of standardized pre-treatment and processing protocols, limits reproducibility and scalability. Regulatory and institutional gaps further constrain industrial adoption, as few targeted incentives or guidelines exist to stimulate circular textile innovation.

We identified a crucial shortcoming: the absence of comprehensive LCA and TEA studies, which are essential for validating environmental impacts, ensuring process sustainability, and informing investment decisions. Consequently, most current research remains confined to the experimental stage, without validation at pilot or industrial scales.

Overcoming these barriers requires coordinated strategies that combine scientific innovation, technological scaling, and policy alignment. Investments in advanced biocomposites, green extraction methods, and standardized testing frameworks are essential to ensure both environmental integrity and economic viability. Achieving a regenerative textile sector depends not only on reusing waste but also on restructuring value chains to integrate renewable materials, low-impact technologies, and long-term financial feasibility.

## 7. Future Research Directions

Scientifically, the main advances have occurred in fiber extraction, bio-based coating development, and alternative leather production, supported by eco-friendly chemical and biological routes. However, significant bottlenecks remain, such as the heterogeneity of agricultural residues, the lack of standardized pre-treatment protocols, and the scarcity of Life Cycle Assessment (LCA) and Techno-Economic Analysis (TEA) studies that can validate industrial scalability.

Future studies must therefore progress from exploratory experimentation to scalable, industry-oriented applications capable of converting agro-industrial residues into functional and competitive textile materials. This requires rigorous assessment of feedstock variability, process optimization, cost–performance relationships, and material stability to ensure that laboratory results are reproducible at pilot and industrial scales. Validation through semi-industrial trials should become a research priority, generating robust datasets for techno-economic and life cycle modeling that can inform decision-making and policy formulation.

Equally important is advancing research on consumer perception and market acceptance. Understanding how end-users evaluate the quality, aesthetics, and performance of bio-based textiles is essential for successful market entry and long-term adoption. Integrating behavioral and socio-economic analyses into technological studies will provide a holistic view that connects innovation with consumer demand.

In parallel, cross-sector collaboration must evolve into coordinated innovation ecosystems involving academia, industry, and policymakers. Such networks are vital for harmonizing methodologies, facilitating technology transfer, and establishing certification and standardization frameworks that substantiate the environmental claims and performance of agro-based textile products.

By addressing these interconnected technical, economic, and social challenges, the scientific community can advance from proof-of-concept experiments to commercially viable, environmentally responsible, and socially accepted solutions. This integrated approach will accelerate the transformation of the textile sector toward a truly circular, low-carbon, and regenerative economy, in full alignment with the United Nations Sustainable Development Goals (SDGs 9, 12, and 13).

## Figures and Tables

**Table 1:** Past ten years of papers addressing the recovery of textile fibers from plant agriwaste.

Fiber Source	Extraction Approach	Main Contributions	Ref.
Banana (*Musa* spp.) stem	Chemical retting, mechanical or manual extraction	In this revision, authors verified that mechanical extraction was the chosen method to recover fibers from the banana stem. Fibers isolated from the inner stem were soft and silk-like, whereas fibers from the outer stem were harsher and less pliable than the first.	[22]
Pineapple [*Ananas comosus* (L.) Merril] leaves	Water retting and degumming	Optimal fiber retting, conducted over 7 days at ambient temperature, achieved a yield of 2.8%. Scanning Electron Microscopy (SEM) analysis confirmed the removal of pectin and leaf matter. The fiber’s physical properties (tensile strength, friction, water absorbency, and surface texture) suggest its potential for high-quality yarn production.	[33]
Corn (*Zea mays* L.) husk	Enzyme retting	A two-step scouring process improved absorbency and whiteness of fibers for better bleaching and dyeing results. Blended yarns of corn husk fibers and coir or wood pulp fiber were developed.	[30,37]
Coconut (*Cocos nucifera* L.) husk	Mechanical extraction or retting	Fine fibers were treated with a mild alkali solution and H_2_O_2_ for scouring and pre-bleaching, and then with a cationic softener for 10 min at 60 °C. Such process was effective in enabling spinning and weaving to a textile material.	[38]
Hemp (*Cannabis sativa* L.) stem	Mechanical extraction and degumming	Low-cost ethanol-assisted degumming diminished fiber degradation. Morphology and composition analyses showed a smooth, high tensile strength and whitened fiber.	[39]
Sugarcane (*Saccharum officinarum* L.) bagasse rind and inner pith	Water and alkali retting variating temperature, time and concentrations of NaOH,	Authors revised fiber recovery reports from sugarcane bagasse, summarizing common points in their routes. Rind-derived fibers were stronger and coarser than other natural fibers. Cellulose and nano cellulose recovery resulted in smaller and weaker fibers than rind-derived ones.	[27]
Kenaf (*Hibiscus cannabinus* L.) stem	Dew retting, water retting, enzyme retting, mechanical retting and acid and alkali retting	Comparative cost analysis revealed that kenaf fibers are potentially cost-effective alternatives to cotton, flax, aramid and other fibers, considering their low cost, low density, medium tensile strength and stiffness. Kenaf fibers or powder enhanced the tensile strength of composite products.	[40,41]
Malva (*Malva sylvestris* L.) stem; curauá (*Ananas erectifolius* L.B. Smith) leaves	Chemical retting	Alkali treatment with NaOH enhanced fibers’ roughness and bonding strength, although it failed in improving fibers’ tensile and flexural strength. Hemicellulose was totally removed from the malva fibers, but partially removed from the curauá fibers, as confirmed by Thermogravimetric (TGA) and SEM analysis.	[42]
Barley (*Hordeum vulgare* L.) straw	Water, dew, enzymes, acid or alkali retting	Recovered fibers had moisture regain higher than cotton fibers and tenacity was enough to endure yarning formation.	[43]
Areca (*Areca catechu* L.) nutshell husk and leaves	Chemical or water retting	Fiber extraction from husk was simple and reproducible. According to the authors, both coarse fibers from husk and weaker fibers from leaves can be applied as yarn, mixed yarns, composites and as alternatives to synthetic fibers.	[44]
Okra [*Abelmoschus esculentus* (L.) Moench] stem	Chemical, due or water retting	In this review, okra stem fibers were featured as holding tensile strength, dyeability, and colorfastness. Bleaching, alkalization, and graft copolymerization improved fibers’ characteristics as color, water absorption and smoothness.	[26]
Tucum (*Bactris setosa* Mart.) leaves	Manual extraction	Unusual palm tree leaflets fibers were extracted in a slow chemical-free process. Recovered fibers were characterized, bleached and dyed. Fiber properties such as length, high tensile strength and dyes absorption were similar to cotton.	[9]
Rice (*Oryza sativa* L.) straw	Water retting	A mixed yarn composed of 12% rice straw, 23% rayon and 65% polyesters was dyed and finished with Starguard FCS to achieve water repellency. The resulting yarn repelled water efficiently and color was maintained, although the resistance to pilling was reduced.	[45]
Canola (*Brassica napus* L.) stem	Water retting or manual extraction	Recovered fibers were light, hydrophilic and suitable for apparel, non-woven, composite and other applications. SEM analysis indicated a low-density bast fiber, corroborating its lightweight and low tensile strength features.	[46]
Mango (*Mangifera indica* L.) residue	Mechanical processing	Mango-starch was cast-tape dried to produce a vegetable leather-like fabric. Mango small-length fibers are suitable for non-woven textiles applications.	[47]

**Table 2:** Past decade has seen experimental papers reporting the obtainment of pigments from plant residues and their applications in dying and printing processes.

Pigment Source	Colorant Obtainment	Color Finishing, Fixation Technique and Outcomes	Reference
Poppy (*Papaver* spp.) flowers and leaves of sycamore (*Platanus occidentalis* L.); Linden (*Tilia* spp.); walnut (*Juglans* spp.) and rose (*Rosa indica* L.)	Mechanical pressure by rolling the fabrics on a tubular draping structure, tying and heating	Ecoprinting cotton fabric and leather using three different mordants: copper sulfate, iron sulfate and spurge plant (biomordant). The fixation and vivid colors of plants and flowers had better outcomes with biomordant application.	[58]
Indian rose leaves (*Rosa indica* L.)	Mechanical pressure by careful hammering, steaming rolled fabrics and post mordanting	Ecoprinting cotton and silk fabric with potassium aluminum sulfate (alum) as mordant. The pigment absorption was fast, easy and highly replicable.	[59]
Mangosteen *(Garcinia mangostana* L.) rind	Rinds were washed, shade dried and powdered	Authors investigated the dyeing outcomes of pretreated leather with the following mordants: calcium carbonate, ferrous sulfate, citric acid, zinc sulphate, chestnut, or aluminum sulphate resulting in different reflectance values and color shades.	[60]
Onion (*Allium cepa* L.) peel extract	Red onion peels were dried, crushed and prepared by decoction	Kanwal et al. (2025) [56] reported that the ultrasonic dyeing of polyester fabric with alum mordant resulted in a light to medium color shade. Islam et al. (2022) [61] compared banana peel and guava leave mordants in dyeing jute-cotton pretreated fabric. The first resulted in better color fixation after washing, whereas the second provided better color shade.	[56,61]
Spent coffee (*Coffea* spp.) grounds and spent black tea (*Camellia sinensis* L.) leaves	Decoction, centrifugation and filtered	Spent black tea leaves’ colorant resulted in a darker color shade and higher antioxidant capacity than the spent coffee’s colorant, possibly due to the tannin and flavonoid components in its leaves.	[62,63]
Coconut (*Cocos nucifera* L.) husk fibers	Fibers were washed, dried and powdered	Cotton fabrics were dyed using alum and vinegar as mordants under varying concentrations and methods. Optimal results were defined with specific dye-mordant combinations, resulting in satisfactory washing and light fastness, as assessed by gray scale evaluation.	[64]
Eucalyptus (*Eucalyptus grandis* W. Hill ex Maiden) wood liquid residue	Lumber steaming resulting in a colored liquid residue (colorant)	Exhaustion dyeing of a cotton fabric with no use of mordants resulting in a low color intensity dyeing. The reported process consumed less water and energy in comparison with dyeing with other plant-based dyes.	[65]
“Sangra-d’água” (*Croton urucurana* Baill) wood bark	Barks were washed, dried and milled	The tannin-rich extract was applied to dye cotton and wool textiles, producing shades ranging from beige to reddish brown. Optimization of the dyeing process was achieved through a 2^3^-factorial experimental design. The dyeing wastewater requires treatment.	[66]
Black beans (*Phaseolus vulgaris* L.) skin	Filtration of wastewater from soaked beans; the obtained anthocyanin extract was directly applied as colorant	Exhaustion wool dyeing with iron and alum tested as mordants resulted in color gain and UV protection. Silk exhaustion dyeing had time, temperature and pH interfering with the color shades. Best conditions for pigmentation on silk was verified for pH 1, at 60 °C for 60 min.	[67]
Beetroot (*Beta vulgaris* L.) peels	Dissolution in water, under pressure or enzyme-assisted extraction	The tree extraction methods were compared to develop a colorant for wool dyeing. An acid environment, chelating agents and temperature variation result in different and possible new colors for textile pigments.	[68]
Black rice (*Oryza sativa* L.) bran extract	Filtration of wastewater from soaked rice grains. The recovered anthocyanin extract was diluted and directly applied	Wool dyeing with metal mordants resulted in brown color changing shades varying according to pH and mordant. The extract antibacterial features were reported.	[32]

**Table 3:** Economic categories and technical feasibility of reusing agro-industrial waste.

Category	Description	Technical Feasibility	Examples and Impacts	References
Collection and transportation	Collection logistics in remote rural areas; adequate storage infrastructure.	Feasible, but requires efficient logistics planning to optimize costs.	Collection of pineapple peels for fiber extraction, promoting agricultural waste reduction and fostering the integration of rural communities.	[97]
Processing technology	Investment in machinery for fiber extraction, chemical processing, and the production of sustainable filaments.	Feasible with already available technologies but requires adaptation for specific materials.	Utilization of extruders for processing coconut shell fibers, enabling the replacement of synthetic fibers and reducing dependence on non-renewable resources.	[98]
Process adaptation	Modifications in textile equipment, including looms and spinning machines, to accommodate recycled or natural fibers derived from waste.	Feasible with technical training and adjustments in existing equipment.	Adaptation of looms for processing banana fibers, enhancing efficiency in the production of sustainable fabrics.	[5]
Research and development	Research focused on developing new materials and optimizing dyeing and finishing processes using agro-industrial waste.	Fundamental for investigating the technical and aesthetic properties of innovative products.	Development of biodegradable fabrics dyed with natural pigments extracted from fruit peels, fostering the creation of premium ecological products.	[99]
Governmental incentives	Tax incentives, subsidies, and specialized credit lines for companies implementing sustainable practices in textile production.	Enhances economic viability while driving the transition to a circular economy.	Incentives for natural dyeing projects, lowering initial costs and fostering a sustainable economy.	[100]
Environmental impacts	Decreased solid waste generation and reduced reliance on synthetic raw materials like polyester.	Highly feasible through clean processes and the integration of agro-industrial waste.	Production of biodegradable fabrics from plant-based fibers, reducing the environmental impact of textile product disposal.	[5,9,101]
Human resources training	Technical training for process adaptation and operation of advanced technologies tailored to the textile industry.	Feasible through partnerships between companies and educational institutions.	Training programs on using recycled fibers in weaving, enhancing workers qualifications and boosting sector competitiveness.	[102]
Marketing and positioning	Investments to showcase the environmental and social advantages of sustainable products in the textile market.	Essential for attracting conscious consumers and solidifying added value.	Development of eco-labels and certifications for recycled fabrics, enabling access to premium markets and boosting exports.	[103]

## Data Availability

The original contributions presented in the study are included in the article, further inquiries can be directed to the corresponding author.

## References

[1] Deshmukh S.M., Dhokpande S.R., Sankhe A., Khandekar A. (2025). Effluent Wastewater Technologies for Textile Industry: A Review. Rev. Inorg. Chem..

[2] Tripathi M., Sharma M., Bala S., Thakur V.K., Singh A., Dashora K., Hart P., Gupta V.K. (2024). Recent Technologies for Transforming Textile Waste into Value-Added Products: A Review. Curr. Res. Biotechnol..

[3] Subramanian K., Sarkar M.K., Wang H., Qin Z.-H., Chopra S.S., Jin M., Kumar V., Chen C., Tsang C.-W., Lin C.S.K. (2022). An Overview of Cotton and Polyester, and Their Blended Waste Textile Valorisation to Value-Added Products: A Circular Economy Approach–Research Trends, Opportunities and Challenges. Crit. Rev. Environ. Sci. Technol..

[4] Clark J.H. (2022). Using Green Chemistry to Progress a Circular Fashion Industry. Curr. Opin. Green Sustain. Chem..

[5] de Oliveira C.R.S., da Silva Júnior A.H., Mulinari J., Immich A.P.S. (2021). Textile Re-Engineering: Eco-Responsible Solutions for a More Sustainable Industry. Sustain. Prod. Consum..

[6] Centobelli P., Abbate S., Nadeem S.P., Garza-Reyes J.A. (2022). Slowing the Fast Fashion Industry: An All-Round Perspective. Curr. Opin. Green Sustain. Chem..

[7] Ikram M. (2022). Transition toward Green Economy: Technological Innovation’s Role in the Fashion Industry. Curr. Opin. Green Sustain. Chem..

[8] Salazar Sandoval S., Amenábar A., Toledo I., Silva N., Contreras P. (2024). Advances in the Sustainable Development of Biobased Materials Using Plant and Animal Waste as Raw Materials: A Review. Sustainability.

[9] Flohr T.T., Neiva E.G.C., Dantas M.P., Corrêa R.C.G., Yamaguchi N.U., Peralta R.M., da Silva Júnior A.H., da Cruz J.A., de Aguiar C.R.L., de Oliveira C.R.S. (2024). Characterization of Atlantic Forest Tucum (*Bactris setosa* Mart.) Leaf Fibers: Aspects of Innovation, Waste Valorization and Sustainability. Plants.

[10] Akatwijuka O., Gepreel M.A.-H., Abdel-Mawgood A., Yamamoto M., Saito Y., Hassanin A.H. (2024). Overview of Banana Cellulosic Fibers: Agro-Biomass Potential, Fiber Extraction, Properties, and Sustainable Applications. Biomass Conv. Bioref..

[11] Phan K., Raes K., Van Speybroeck V., Roosen M., De Clerck K., De Meester S. (2021). Non-Food Applications of Natural Dyes Extracted from Agro-Food Residues: A Critical Review. J. Clean. Prod..

[12] Saba B., Christy A.D., Jabeen M. (2016). Kinetic and Enzymatic Decolorization of Industrial Dyes Utilizing Plant-Based Biosorbents: A Review. Environ. Eng. Sci..

[13] Dahiya D., Nigam P.S. (2020). Waste Management by Biological Approach Employing Natural Substrates and Microbial Agents for the Remediation of Dyes’ Wastewater. Appl. Sci..

[14] Gaminian H., Ahvazi B., Vidmar J.J., Ekuere U., Regan S. (2024). Revolutionizing Sustainable Nonwoven Fabrics: The Potential Use of Agricultural Waste and Natural Fibres for Nonwoven Fabric. Biomass.

[15] Plakantonaki S., Kiskira K., Zacharopoulos N., Belessi V., Sfyroera E., Priniotakis G., Athanasekou C. (2024). Investigating the Routes to Produce Cellulose Fibers from Agro-Waste: An Upcycling Process. ChemEngineering.

[16] FAO (2024). World Food and Agriculture–Statistical Yearbook 2024.

[17] FAO (2024). Pesticides Use and Trade, 1990–2022.

[18] FAO (2024). Inorganic Fertilizers.

[19] FAO (2024). Land Statistics 2001–2022.

[20] FAOSTAT (2024). Employment Indicators 2000–2022.

[21] FAO (2024). Food and Agriculture Organization of the United Nations. FAOSTAT Analytical Brief 96–Agricultural Production Statistics 2010–2023.

[22] Jayaprakash K., Osama A., Rajagopal R., Goyette B., Karthikeyan O.P. (2022). Agriculture Waste Biomass Repurposed into Natural Fibers: A Circular Bioeconomy Perspective. Bioengineering.

[23] Bhatia T., Sindhu S.S. (2024). Sustainable Management of Organic Agricultural Wastes: Contributions in Nutrients Availability, Pollution Mitigation and Crop Production. Discov. Agric..

[24] OECD (2019). FAO Background Notes on Sustainable, Productive and Resilient Agro-Food Systems: Value Chains, Human Capital, and the 2030 Agenda.

[25] United Nations Transforming Our World (2015). The 2030 Agenda for Sustainable Development.

[26] Gupta P.K., Patra S., Samanta K.K. (2021). Potential of Okra for Application in Textiles: A Review. J. Nat. Fibers.

[27] Mahmud M.A., Anannya F.R. (2021). Sugarcane Bagasse–A Source of Cellulosic Fiber for Diverse Applications. Heliyon.

[28] da Silva J.J., da Silva B.F., Stradiotto N.R., Petrović M., Gros M., Gago-Ferrero P. (2021). Identification of Organic Contaminants in Vinasse and in Soil and Groundwater from Fertigated Sugarcane Crop Areas Using Target and Suspect Screening Strategies. Sci. Total Environ..

[29] FAOSTAT Crops and Livestock Products. https://www.fao.org/faostat/en/#data/QCL/visualize.

[30] Patil H., Athalye A. (2023). Valorization of Corn Husk Waste for Textile Applications. J. Nat. Fibers.

[31] Borah M.P., Jose S., Kalita B.B., Shakyawar D., Pandit P. (2020). Water Repellent Finishing on Eri Silk Fabric Using Nano Silica. J. Text. Inst..

[32] Haque M.A., Mia R., Mahmud S.T., Bakar M.A., Ahmed T., Farsee M.S., Hossain M.I. (2022). Sustainable Dyeing and Functionalization of Wool Fabrics with Black Rice Extract. Resour. Environ. Sustain..

[33] Hazarika D., Gogoi N., Jose S., Das R., Basu G. (2017). Exploration of Future Prospects of Indian Pineapple Leaf, an Agro Waste for Textile Application. J. Clean. Prod..

[34] Rosa F.R., Arruda A.F., Siqueira E.M.A., Arruda S.F. (2016). Phytochemical Compounds and Antioxidant Capacity of Tucum-Do-Cerrado (Bactris Setosa Mart), Brazil’s Native Fruit. Nutrients.

[35] da Fonseca R.P., Rocha J.C., Cheriaf M. (2021). Mechanical Properties of Mortars Reinforced with Amazon Rainforest Natural Fibers. Materials.

[36] Unuofin J.O., Moloantoa K.M., Khetsha Z.P. (2022). The Biobleaching Potential of Laccase Produced from Mandarin Peelings: Impetus for a Circular Bio-Based Economy in Textile Biofinishing. Arab. J. Chem..

[37] Jain A., Rastogi D., Chanana B. (2018). Utilization of Cornhusk for Textile Usages. J. Basic Appl. Eng. Res..

[38] Martins A.P., Sanches R.A. (2019). Assessment of Coconut Fibers for Textile Applications. Matéria.

[39] Lyu P., Xia L., Jiang X., Liu X., Xu W., Hurren C., Wang X. (2022). Efficient Extraction of Technical Fibers from Hemp in an Ethanol-Water Mixture. Ind. Crops Prod..

[40] Sreenivas H.T., Krishnamurthy N., Arpitha G.R. (2020). A Comprehensive Review on Light Weight Kenaf Fiber for Automobiles. Int. J. Lightweight Mater. Manuf..

[41] Shahar F.S., Sultan M.T.H., Shah A.U.M., Safri S.N.A. (2019). A Short Review on the Extraction of Kenaf Fibers and the Mechanical Properties of Kenaf Powder Composites. IOP Conf. Ser. Mater. Sci. Eng..

[42] Sankar K., Constâncio Trindade A.C., Kriven W.M. (2023). The Influence of Alkaline Treatment on the Mechanical Performance of Geopolymer Composites Reinforced with Brazilian Malva and Curaua Fibers. J. Am. Ceram. Soc..

[43] Kovačević Z., Strgačić S., Bischof S. (2023). Barley Straw Fiber Extraction in the Context of a Circular Economy. Fibers.

[44] Sunny G., Rajan T.P. (2022). Review on Areca Nut Fiber and Its Implementation in Sustainable Products Development. J. Nat. Fibers.

[45] Jatuphatwarodom S., Jatuphatwarodom N., Susawat K. Dyeing and Water Repellent Finishing of Thai Rice Straw Blended Fabric for Home Textile Products. Proceedings of the RSU International Research Conference.

[46] Shuvo I.I., Rahman M., Vahora T., Morrison J., DuCharme S., Choo-Smith L.-P. (2020). Producing Light-Weight Bast Fibers from Canola Biomass for Technical Textiles. Text. Res. J..

[47] da Silva Simão R., de Moraes J.O., de Souza P.G., Mattar Carciofi B.A., Laurindo J.B. (2019). Production of Mango Leathers by Cast-Tape Drying: Product Characteristics and Sensory Evaluation. LWT.

[48] García C., Prieto M.A. (2019). Bacterial Cellulose as a Potential Bioleather Substitute for the Footwear Industry. Microb. Biotechnol..

[49] Provin A.P., de Dutra A.R.A., de Sousa e Silva Gouveia I.C.A., Cubas E.A.L.V. (2021). Circular Economy for Fashion Industry: Use of Waste from the Food Industry for the Production of Biotextiles. Technol. Forecast. Soc. Change.

[50] Zhang A.-N., Zhao H.-B., Cheng J.-B., Li M.-E., Li S.-L., Cao M., Wang Y.-Z. (2021). Construction of Durable Eco-Friendly Biomass-Based Flame-Retardant Coating for Cotton Fabrics. Chem. Eng. J..

[51] Deng M., Zhang G., Zeng Y., Pei X., Huang R., Lin J. (2016). Simple Process for Synthesis of Layered Sodium Silicates Using Rice Husk Ash as Silica Source. J. Alloys Compd..

[52] Ren Y., Liu Y., Wang Y., Guo X., Liu X. (2020). Preparation of Durable and Flame Retardant Lyocell Fabrics by Using a Biomass-Based Modifier Derived from Vitamin C. Cellulose.

[53] Ariram N., Madhan B. (2020). Development of Bio-Acceptable Leather Using Bagasse. J. Clean. Prod..

[54] Baraldi R.F.D.S., Neiva E.C., Da Silva Júnior A.H., Costa T.M., Gonçalves M.J., De Aguiar C.L., Nihues T.C., Schlindwein R., Missner M.E.P., De Oliveira C.R.S. (2025). Bio-Based Flame Retardant for Cotton Fabric Prepared from Eggshell Microparticles, Phytic Acid, and Chitosan: An Eco-Friendly Approach for Dry Use. Processes.

[55] (2019). Standard Methods of Fire Tests for Flame Propagation of Textiles and Films.

[56] Kanwal R., Hashim M., Naz S., Malik S.A., Mengal N., Ashraf R.F., Mansour Y., Subramaniam U., Mustaffa Z., Abdelhadi A., Ezzat M., Abowardah E. (2025). Sustainable Coloration of Polyester Fabric Using Onion Peel Extract with Ultrasonic Energy. Proceedings of the International Conference on Sustainability: Developments and Innovations.

[57] Jordeva S., Kertakova M., Zhezhova S., Golomeova L.S., Mojsov K. (2020). Dyeing of Textiles with Natural Dyes. Tekst. Ind..

[58] Çolak S., Arğun F.N., Kaygusuz M. (2021). Fashionable Leather Products From Ecofriendly Desinged Vegetable Tanned Leathers. Motif Akad. Halkbilim Derg..

[59] Manuja A., Ojha I., Singh D.M. (2023). Eco-Printing: Domestic Technique of Textile Printing Using the Leaves of Rose Indica. IJETMS.

[60] Kurinjimalar C., Usharani N., Kanimozhi B., Jayakumar G.C., Kanth S.V. (2022). Extraction and Optimization of Natural Colorant from *Garcinia mangostana* Linn Peels for Leather Dyeing. Clean. Eng. Technol..

[61] Islam M.R., Khan A.N.N., Mahmud R.U., Haque S.M.N., Khan M.M.I. (2022). Sustainable Dyeing of Jute-Cotton Union Fabrics with Onion Skin (*Allium CEPA*) Dye Using Banana Peel (*Musa*) and Guava Leaves (*Psidium guajava*) Extract as Biomordants. Pigment Resin Technol..

[62] Xia W., Li Z., Tang Y., Li Q. (2023). Sustainable Recycling of Café Waste as Natural Bio Resource and Its Value Adding Applications in Green and Effective Dyeing/Bio Finishing of Textile. Sep. Purif. Technol..

[63] Sukemi, Pratumyot K., Srisuwannaket C., Niamnont N., Mingvanish W. (2019). Dyeing of Cotton with the Natural Dye Extracted from Waste Leaves of Green Tea (*Camellia sinensis* var. *assamica*). Color. Technol..

[64] Kashyap R., Sharma N., Sharma L. (2016). Divya Dyeing of Cotton with Natural Dye Extract from Coconut Husk. Int. J. Sci. Technol. Eng..

[65] Rossi T., Silva P.M.S., De Moura L.F., Araújo M.C., Brito J.O., Freeman H.S. (2017). Waste from Eucalyptus Wood Steaming as a Natural Dye Source for Textile Fibers. J. Clean. Prod..

[66] dos Santos Silva P.M., Fiaschitello T.R., de Queiroz R.S., Freeman H.S., da Costa S.A., Leo P., Montemor A.F., da Costa S.M. (2020). Natural Dye from *Croton urucurana* Baill. Bark: Extraction, Physicochemical Characterization, Textile Dyeing and Color Fastness Properties. Dye. Pigment..

[67] Punyachareonnon P., Deerattrakul V., Luepong K. (2021). The Influence of pH, Temperature and Time on Dyeing of Silk Fabric by Black Bean Anthocyanin-Rich Extract as Colorant. Prog. Color Color. Coat..

[68] Popescu V., Blaga A.C., Pruneanu M., Cristian I.N., Pîslaru M., Popescu A., Rotaru V., Crețescu I., Cașcaval D. (2021). Green Chemistry in the Extraction of Natural Dyes from Colored Food Waste, for Dyeing Protein Textile Materials. Polymers.

[69] Lee Park C., Fracarolli Nunes M. (2024). Vegan Luxury for Non-Vegan Consumers: Impacts on Brand Trust and Attitude towards the Firm. J. Retail. Consum. Serv..

[70] Muthu S.S., Ramchandani M., Muthu S.S., Ramchandani M. (2024). Natural/Agro-Derived Versus Artificial Vegan Leather: How Leather Alternatives Influence the Sustainable Luxury and Fashion Industry. Vegan Alternatives for Leather.

[71] Wijayarathna E.R.K.B., Svensson S.E., Sar T., Zamani A. (2025). Multilayer Biocomposite Vegan Leather Materials Derived from Vegetable-Tanned Fungal Biomass Cultivated on Food Waste. Sci. Rep..

[72] Patil H., Patil Y., Maiti S., Athalye A., Adivarekar R.V. (2024). Valorization of Fruit Vegetable Waste for Semi-Synthetic Leather. Iran Polym. J..

[73] Patil K.K.R., Shashidara K.C., Sowmya H.S., Suresh S.R. (2024). Vegan Areca Palm Leather–Waste to Wealth Generation through Agri-Start-Up. Curr. Sci..

[74] Mecca S., Digiovanni S., Milanesi R., Frigerio C., Mangiagalli M., Tarricone G., Boventi M., Bordignon S., Clerici M., Lotti M. (2025). Development of Leather-like Materials from Enzymatically Treated Green Kiwi Peel and Valorization of By-Products for Microbial Bioprocesses. ACS Sustain. Chem. Eng..

[75] Rimantho D., Chaerani L., Sundari A.S. (2024). Initial Mechanical Properties of Orange Peel Waste as Raw Material for Vegan Leather Production. Case Stud. Chem. Environ. Eng..

[76] Liu X., Zhang X., Wang X., Yue O., Jiang H. (2025). Engineered, Environmentally Friendly Leather-like Bio-Based Materials. Trends Biotechnol..

[77] Fuad S.M., Farmer M.C., Adisa A. (2025). Economic Opportunities of Bioelectricity from Cotton Gin Waste. J. Agric. Appl. Econ..

[78] Shah M.A., Hayder G., Kumar R., Kumar V., Ahamad T., Kalam M.A., Soudagar M.E.M., Mohamed Shamshuddin S.Z., Mubarak N.M. (2023). Development of Sustainable Biomass Residues for Biofuels Applications. Sci. Rep..

[79] Zheng N.-Y., Lee M., Lin Y.-L. (2020). Co-Processing Textile Sludge and Lignocellulose Biowaste for Biofuel Production through Microwave-Assisted Wet Torrefaction. J. Clean. Prod..

[80] Xu Y., Su Q., Shen H., Xu G. (2019). Physicochemical and Sorption Characteristics of Poplar Seed Fiber as a Natural Oil Sorbent. Text. Res. J..

[81] Wu J., Yang J., Huang G., Xu C., Lin B. (2020). Hydrothermal Carbonization Synthesis of Cassava Slag Biochar with Excellent Adsorption Performance for Rhodamine B. J. Clean. Prod..

[82] Rahman M.M., Maniruzzaman M., Mahmud P., Khatun S., Hossain M.K., Hossain M.I., Hossain M.I., Hasanuzzaman M., Alam M.A., Al-amin M. (2025). Adsorptive Removal of Toxic Heavy Metal and Dyes from Wastewater by Rice Husk (*Lignocellulosic biomass*) Derived Activated Biochar: A Fixed-Bed Column Adsorption Study. Carbohydr. Polym. Technol. Appl..

[83] Vasconcelos W.M., Mulinari J., Leal T.W., de Oliveira C.R.S., da Silva Júnior A.H., Lourenço L.A., Lenzi C. (2025). Exploring Pristine Tucumã (*Astrocaryum Aculeatum*) Waste as a Sustainable Adsorbent for Textile Dye Removal. Environ. Qual. Manag..

[84] Prado-Acebo I., Cubero-Cardoso J., Lu-Chau T.A., Eibes G. (2024). Integral Multi-Valorization of Agro-Industrial Wastes: A Review. Waste Manag..

[85] Kamboj A., Sadh P.K., Yadav B., Kumari A., Kumar R., Surekha, Saharan B.S., Brar B., Kumar D., Goyal C. (2024). Unravelling the Potential of Sugarcane Bagasse: An Eco-Friendly and Inexpensive Agro-Industrial Waste for the Production of Valuable Products Using Pretreatment Processes for Sustainable Bio-Economy. J. Environ. Chem. Eng..

[86] Chatterjee B., Mazumder D. (2024). A Critical Review of the Advances in Valorizing Agro-Industrial Wastes through Mixed Culture Fermentation. J. Environ. Chem. Eng..

[87] Wang Y., Wang Z., Lin Y., Qin Y., He R., Wang M., Sun Q., Peng Y. (2024). Nanocellulose from Agro-Industrial Wastes: A Review on Sources, Production, Applications, and Current Challenges. Food Res. Int..

[88] Phiri R., Mavinkere Rangappa S., Siengchin S. (2024). Agro-Waste for Renewable and Sustainable Green Production: A Review. J. Clean. Prod..

[89] Wagh M.S., Sowjanya S., Nath P.C., Chakraborty A., Amrit R., Mishra B., Mishra A.K., Mohanta Y.K. (2024). Valorisation of Agro-Industrial Wastes: Circular Bioeconomy and Biorefinery Process–A Sustainable Symphony. Process Saf. Environ. Prot..

[90] Rame R., Purwanto P., Sudarno S. (2023). Biotechnological Approaches in Utilizing Agro-Waste for Biofuel Production: An Extensive Review on Techniques and Challenges. Bioresour. Technol. Rep..

[91] Verma P., Kaushik R., Sirohi R. (2024). A Review on the Preparation, Characterization, and Applications of Agro-Waste-Derived Oligosaccharides. Food Biosci..

[92] de Azevedo A.R.G., Amin M., Hadzima-Nyarko M., Saad Agwa I., Zeyad A.M., Tayeh B.A., Adesina A. (2022). Possibilities for the Application of Agro-Industrial Wastes in Cementitious Materials: A Brief Review of the Brazilian Perspective. Clean. Mater..

[93] Lima C.A., Bento H.B.S., Picheli F.P., Paz-Cedeno F.R., Mussagy C.U., Masarin F., Torres Acosta M.A., Santos-Ebinuma V.C. (2023). Process Development and Techno-Economic Analysis of Co-Production of Colorants and Enzymes Valuing Agro-Industrial Citrus Waste. Sustain. Chem. Pharm..

[94] Mulinari J., Junior F.W.R., de Oliveira C.R.S., da Silva Júnior A.H., Scariot M.A., Radünz L.L., Mossi A.J., Thapar Kapoor R., Treichel H., Shah M.P. (2021). Biochar as a Tool for the Remediation of Agricultural Soils. Biochar and Its Application in Bioremediation.

[95] Ramaiah G., Simeno Z., Negawo T.A., Baraki S.Y., Legese R., Asfaw D. (2025). Extraction of Ensete Fibers and Its Woven Fabric Green Composite Development for Ceiling Board Applications. Ind. Crops Prod..

[96] Wang B., Li Z., Wang Y., Zhang B., Lv C., Bi X., Zhao T. (2025). Eco-Friendly Dyeing of Cotton Fabrics with Microbial Pigments: Anionic Modification for Superior Color, Antibacterial, Hydrophobic and UV Protection Properties. Ind. Crops Prod..

[97] Umesh M., Suresh S., Santosh A.S., Prasad S., Chinnathambi A., Al Obaid S., Jhanani G.K., Shanmugam S. (2023). Valorization of Pineapple Peel Waste for Fungal Pigment Production Using Talaromyces Albobiverticillius: Insights into Antibacterial, Antioxidant and Textile Dyeing Properties. Environ. Res..

[98] Siddiqui M.A.S., Rabbi M.S., Ahmed R.U., Billah M.M. (2024). Biodegradable Natural Polymers and Fibers for 3D Printing: A Holistic Perspective on Processing, Characterization, and Advanced Applications. Clean. Mater..

[99] Gupta N., Paul J.S., Jadhav S.K. (2024). Biovalorizing Agro-Waste ‘de-Oiled Rice Bran’ for Thermostable, Alkalophilic and Detergent Stable α-Amylase Production with Its Application as Laundry Detergent Additive and Textile Desizer. Int. J. Biol. Macromol..

[100] Liu Y., Zhao H., Li X. (2024). Environmental Policy Effects of the Carbon Tax, Subsidy, and Policy Combinations of China’s Textile Industry: Evidence from the DSGE Model. J. Clean. Prod..

[101] Nesterov D., Barrera-Martínez I., Martínez-Sánchez C., Sandoval-González A., Bustos E. (2024). Approaching the Circular Economy: Biological, Physicochemical, and Electrochemical Methods to Valorize Agro-Industrial Residues, Wastewater, and Industrial Wastes. J. Environ. Chem. Eng..

[102] Karanikas N., Hasan S.M.T. (2022). Occupational Health & Safety and Other Worker Wellbeing Areas: Results from Labour Inspections in the Bangladesh Textile Industry. Saf. Sci..

[103] Ghasemy Yaghin R. (2020). Enhancing Supply Chain Production-Marketing Planning with Geometric Multivariate Demand Function (a Case Study of Textile Industry). Comput. Ind. Eng..

[104] Chhandama M.V.L., Chetia A.C., Satyan K.B., Supongsenla A., Ruatpuia J.V., Rokhum S.L. (2022). Valorisation of Food Waste to Sustainable Energy and Other Value-Added Products: A Review. Bioresour. Technol. Rep..

[105] Withanage S.V., Dias G.M., Habib K. (2021). Review of Household Food Waste Quantification Methods: Focus on Composition Analysis. J. Clean. Prod..

[106] Mohanta Y.K., Mishra A.K., Lakshmayya N.S.V., Panda J., Thatoi H., Sarma H., Rustagi S., Baek K.-H., Mishra B. (2025). Agro-Waste-Derived Bioplastics: Sustainable Innovations for a Circular Economy. Waste Biomass Valor..

[107] Butturi M.A., Neri A., Mercalli F., Gamberini R. (2025). Sustainability-Oriented Innovation in the Textile Manufacturing Industry: Pre-Consumer Waste Recovery and Circular Patterns. Environments.

[108] Farhana K., Kadirgama K., Mahamude A.S.F., Mica M.T. (2022). Energy Consumption, Environmental Impact, and Implementation of Renewable Energy Resources in Global Textile Industries: An Overview towards Circularity and Sustainability. Mater. Circ. Econ..

[109] Panhwar A., Sattar Jatoi A., Ali Mazari S., Kandhro A., Rashid U., Qaisar S. (2024). Water Resources Contamination and Health Hazards by Textile Industry Effluent and Glance at Treatment Techniques: A Review. Waste Manag. Bull..

[110] Andrade Siqueira T.C., Zanette da Silva I., Rubio A.J., Bergamasco R., Gasparotto F., Aparecida de Souza Paccola E., Ueda Yamaguchi N. (2020). Sugarcane Bagasse as an Efficient Biosorbent for Methylene Blue Removal: Kinetics, Isotherms and Thermodynamics. Int. J. Environ. Res. Public Health.

[111] Wang X., Li C., Liu K., Zhu L., Song Z., Li D. (2020). Atmospheric Microplastic over the South China Sea and East Indian Ocean: Abundance, Distribution and Source. J. Hazard. Mater..

[112] Xu C., Zhang B., Gu C., Shen C., Yin S., Aamir M., Li F. (2020). Are We Underestimating the Sources of Microplastic Pollution in Terrestrial Environment?. J. Hazard. Mater..

[113] UNEP (2024). Food Waste Index Report 2024. Think Eat Save: Tracking Progress to Halve Global Food Waste.

[114] Sagar N.A., Pathak M., Sati H., Agarwal S., Pareek S. (2024). Advances in Pretreatment Methods for the Upcycling of Food Waste: A Sustainable Approach. Trends Food Sci. Technol..

[115] United Nations World Population Prospects. https://population.un.org/wpp/graphs?loc=900&type=Demographic%20Profiles&category=Line%20Charts.

[116] Clark J.H., Farmer T.J., Herrero-Davila L., Sherwood J. (2016). Circular Economy Design Considerations for Research and Process Development in the Chemical Sciences. Green Chem..

[117] Nájera-Martínez E.F., Melchor-Martínez E.M., Sosa-Hernández J.E., Levin L.N., Parra-Saldívar R., Iqbal H.M.N. (2022). Lignocellulosic Residues as Supports for Enzyme Immobilization, and Biocatalysts with Potential Applications. Int. J. Biol. Macromol..

[118] Sakao T., Bocken N., Nasr N., Umeda Y. (2024). Implementing Circular Economy Activities in Manufacturing for Environmental Sustainability. CIRP Ann..

[119] Mak T.M.W., Xiong X., Tsang D.C.W., Yu I.K.M., Poon C.S. (2020). Sustainable Food Waste Management towards Circular Bioeconomy: Policy Review, Limitations and Opportunities. Bioresour. Technol..

[120] Awasthi M.K., Azelee N.I.W., Ramli A.N.M., Rashid S.A., Manas N.H.A., Dailin D.J., Illias R.M., Rajagopal R., Chang S.W., Zhang Z. (2022). Microbial Biotechnology Approaches for Conversion of Pineapple Waste in to Emerging Source of Healthy Food for Sustainable Environment. Int. J. Food Microbiol..

[121] Guo Z., Yan N., Lapkin A.A. (2019). Towards Circular Economy: Integration of Bio-Waste into Chemical Supply Chain. Curr. Opin. Chem. Eng..

[122] Alan H., Köker A.R. (2023). Analyzing and Mapping Agricultural Waste Recycling Research: An Integrative Review for Conceptual Framework and Future Directions. Resour. Policy.

[123] MacArthur E. (2017). Foundation A New Textiles Economy.

[124] Rana P., Sethi S. (2025). Utilising Agro-Waste in Textile Industry: Advancing Sustainability with a Year-Round Waste Cycle. Mater. Circ. Econ..

